# Evidence for chronic, peripheral activation of neutrophils in polyarticular juvenile rheumatoid arthritis

**DOI:** 10.1186/ar2048

**Published:** 2006-09-26

**Authors:** James N Jarvis, Howard R Petty, Yuhong Tang, Mark Barton Frank, Philippe A Tessier, Igor Dozmorov, Kaiyu Jiang, Andrei Kindzelski, Yanmin Chen, Craig Cadwell, Mary Turner, Peter Szodoray, Julie L McGhee, Michael Centola

**Affiliations:** 1 Department of Pediatrics, University of Oklahoma College of Medicine, 940 Stanton L. Young Blvd., Oklahoma City, OK 73104, USA; 2 Kellogg Eye Center, University of Michigan School of Medicine, 1000 Wall St., Ann Arbor, MI 48105, USA; 3 Arthritis & Immunology Program, Oklahoma Medical Research Foundation, 820 NE 13^th ^St., Oklahoma City, OK 73104, USA; 4 Centre de Recherche en Infectiologie, Centre de Recherche du CHUL, 2705 boul. Laurier, Ste-Foy, Québec, G1V 4G2, Canada; 5 University of Oklahoma College of Medicine, 940 Stanton L. Young Blvd., Oklahoma City, OK 73104, USA

## Abstract

Although strong epidemiologic evidence suggests an important role for adaptive immunity in the pathogenesis of polyarticular juvenile rheumatoid arthritis (JRA), there remain many aspects of the disease that suggest equally important contributions of the innate immune system. We used gene expression arrays and computer modeling to examine the function in neutrophils of 25 children with polyarticular JRA. Computer analysis identified 712 genes that were differentially expressed between patients and healthy controls. Computer-assisted analysis of the differentially expressed genes demonstrated functional connections linked to both interleukin (IL)-8- and interferon-γ (IFN-γ)-regulated processes. Of special note is that the gene expression fingerprint of children with active JRA remained essentially unchanged even after they had responded to therapy. This result differed markedly from our previously reported work, in which gene expression profiles in buffy coats of children with polyarticular JRA reverted to normal after disease control was achieved pharmacologically. These findings suggest that JRA neutrophils remain in an activated state even during disease quiescence. Computer modeling of array data further demonstrated disruption of gene regulatory networks in clusters of genes modulated by IFN-γ and IL-8. These cytokines have previously been shown to independently regulate the frequency (IFN-γ) and amplitude (IL-8) of the oscillations of key metabolites in neutrophils, including nicotinamide adenine dinucleotide (phosphate) (NAD(P)H) and superoxide ion. Using real-time, high-speed, single-cell photoimaging, we observed that 6/6 JRA patients displayed a characteristic defect in 12% to 23% of the neutrophils tested. Reagents known to induce only frequency fluctuations of NAD(P)H and superoxide ion induced both frequency and amplitude fluctuations in JRA neutrophils. This is a novel finding that was observed in children with both active (*n *= 4) and inactive (*n *= 2) JRA. A subpopulation of polyarticular JRA neutrophils are in a chronic, activated state, a state that persists when the disease is well controlled pharmacologically. Furthermore, polyarticular JRA neutrophils exhibit an intrinsic defect in the regulation of metabolic oscillations and superoxide ion production. Our data are consistent with the hypothesis that neutrophils play an essential role in the pathogenesis of polyarticular JRA.

## Introduction

The term juvenile rheumatoid arthritis (JRA) identifies a heterogeneous family of disorders that share the common feature of chronic inflammation and hyperplasia of the synovial membranes. The pathogenesis of JRA is unknown. The histopathologies of adult and juvenile forms of rheumatoid arthritis are identical, suggesting common pathogenic mechanisms. Current theories of disease pathogenesis originate from two key observations: (a) the presence of CD4^+ ^T lymphocytes demonstrating a CD45RO^+ ^('memory') phenotype in inflamed synovium and (b) the strong association of specific HLA (human leukocyte antigen) class II alleles with disease risk for specific JRA subtypes [1]. These two observations have been the foundation of the widely accepted theory that JRA pathogenesis is linked to disordered regulation of T-cell function. According to this hypothesis, the presence of antigen within the synovium is the initiating factor leading to the 'homing' of antigen-specific T cells to the site of antigen deposition (that is, the synovial tissue and fluid).

However, T cell-based hypotheses do not easily account for the well-documented inflammatory aspects of JRA, which include complement activation [2], immune complex accumulation [3,4], monocyte secretion of tumour necrosis factor-α (TNF-α) and interleukin (IL)-1β [5], and the predominance of neutrophils in the synovial fluid [6]. These findings point toward an important role of innate immune cells, particularly neutrophils, in this disease. Hence, we have proposed that the pathogenesis of JRA involves complex interactions between innate and adaptive immune systems [7].

Neutrophils are known to contribute to rheumatoid arthritis pathogenesis by the release of oxygen radicals and tissue-degrading enzymes, which can lead to the degradation of the articular cartilage [8]. The potential involvement of neutrophils in JRA pathogenesis has not been well characterised, despite the fact that neutrophils are the most abundant cells within JRA synovial fluids [6]. However, new data suggest that neutrophils may indeed play an important role in JRA and that neutrophil activation products may serve as biomarkers of disease activity [9]. We used genome-scale expression profiling to examine neutrophil function in children with polyarticular onset JRA, specifically testing the hypothesis that chronic, peripheral neutrophil activation is a characteristic feature of the disease.

## Materials and methods

### Study subjects

We studied 25 children newly diagnosed with rheumatoid factor-negative, polyarticular JRA. Diagnosis was based on accepted and validated criteria endorsed by the American College of Rheumatology (ACR) [10]. Children were excluded if they had been treated with corticosteroids or methotrexate, or if they had received therapeutic doses of nonsteroidal anti-inflammatory drugs for more than 3 weeks prior to study. Patients with active disease ranged in age from 4 to 15 years and presented with proliferative synovitis of multiple joints. All had joint activity scores of at least 15 using a standard scoring system [11] based on that used in pediatric rheumatology clinical trials [12]. Children followed longitudinally were designated as having a 'partial response' to therapy if they met American College of Rheumatology-30 improvement criteria from their baseline state. Children were designated to have inactive disease if there was no objective synovitis on exam, morning stiffness for not more than 20 minutes/day, and a normal erythrocyte sedimentation rate. In addition, we studied 14 of these children on more than one occasion to observe changes in gene expression pattern in response to therapy. S100A8/A9 protein levels, a marker of neutrophil-endothelial cell interactions (see below), were studied in 24 children, 20 of whom were studied on more than one occasion to observe responses to therapy.

Healthy control subjects (*n *= 10) were young adults (age 18 to 30) with no history of rheumatic or chronic inflammatory disease. Previously published work from our group [13] has demonstrated that such subjects are appropriate controls for gene expression studies in children with polyarticular JRA because gene expression profiles of peripheral blood buffy coats of children with polyarticular JRA revert toward patterns indistinguishable from such healthy controls after treatment.

### Sample preparation and RNA purification

After the execution of the informed consent process as approved by the Oklahoma University Health Sciences Center (OUHSC) Institutional Review Board, whole blood (20 cc) was drawn into sterile sodium citrate tubes containing a cell density gradient (cat no. 362761; BD Biosciences, San Jose, CA, USA) and carried immediately to the Pediatric Rheumatology Research laboratories on the OUHSC campus. Granulocytes were immediately separated from mononuclear cells by density gradient centrifugation. Centrifugation was performed at room temperature, resulting in the red cells and granulocytes' layering in the bottom of the tube. Red cells were removed from the granulocytes by hypotonic cell lysis as recommended by the manufacturer, and granulocytes were placed immediately in Trizol reagent for RNA purification. Plasma was removed and stored at -80°C until used in enzyme-linked immunosorbent assays (ELISAs) for S100 protein levels (see below). Cells prepared in this fashion are more than 98% CD66b^+ ^by flow cytometry and contain no contaminating CD14^+ ^cells. Granulocytes were immediately placed in Trizol reagent (Invitrogen, Carlsbad, CA, USA), and RNA was purified exactly as recommended by the manufacturer. RNA was stored under ethanol at -80°C until used for hybridisation and labeling.

### Gene expression arrays

The arrays used in these experiments were developed at the Oklahoma Medical Research Foundation Microarray Core Facility in collaboration with QIAGEN Operon (Alameda, CA, USA). Microarrays were produced using commercially available libraries of 70-nucleotide-long DNA molecules whose length and sequence specificity were optimised to reduce the cross-hybridisation problems encountered with cDNA-based microarrays. The microarrays had 21,329 human genes represented. The oligonucleotides were derived from the UniGene and RefSeq databases. For the genes present in this database, information on gene function, chromosomal location, and reference naming are available. All 11,000 human genes of known or suspected function were represented on these arrays. In addition, most undefined open reading frames were represented (approximately 10,000 additional genes).

Oligonucleotides were spotted onto Corning^® ^UltraGAPS™ amino-silane-coated slides (Acton, MA, USA), rehydrated with water vapor, snap-dried at 90°C, and then covalently fixed to the surface of the glass using 300-mJ, 254-nm wavelength UV radiation. Unbound free amines on the glass surface were blocked for 15 minutes with moderate agitation in a 143 mM solution of succinic anhydride dissolved in 1-methyl-2-pyrolidinone, 20 mM sodium borate, pH 8.0. Slides were rinsed for 2 minutes in distilled water, immersed for 1 minute in 95% ethanol, and dried with a stream of nitrogen gas.

### RNA labeling and hybridization

Prior to cDNA synthesis, the RNA was resuspended in diethylpyrocarbonate-treated water. RNA integrity was assessed using capillary gel electrophoresis (Agilent 2100 BioAnalyzer; Agilent Technologies, Inc., Palo Alto, CA, USA) to determine the ratio of 28 s/18 s rRNA in each sample. A threshold of 1.0 was used to define samples of sufficient quality, and only samples above this limit were used for microarray studies. cDNA was synthesised using Omniscript reverse transcriptase (Qiagen, Valencia, CA, USA) with direct incorporation of cyanine 3-dUTP (deoxy-uridine triphosphate) from 2 μg of RNA. Labeled cDNA was purified using a Montage 96-well vacuum system (Millipore Corporation, Billerica, MA). The cDNA was added to hybridisation buffer containing CoT-1 DNA (0.5 mg/ml final concentration), yeast tRNA (0.2 mg/ml), and poly(dA)_40–60 _(0.4 mg/ml). Hybridisation was performed in an automated liquid delivery, air-vortexed, hybridisation station for 9 hours at 58°C under an oil-based coverslip (Ventana Medical Systems, Inc., Tucson, AZ, USA). Microarrays were washed at a final stringency of 0.1 × SSC (saline-sodium citrate). Microarrays were scanned using a simultaneous dual-colour, 48-slide scanner (Agilent Technologies, Inc.). Fluorescent intensity was quantified using Koadarray™ software (Koada Technology, Kippen, Sterling, UK).

### Array analysis

Data were subject to normalisation and regression steps as described in detail in our earlier work [13]. Genes differentially expressed between groups of samples were selected using associative analysis [13]. Genes selected to be differentially expressed in any sample combinations were used to classify patients, including active, partial and inactive, and control samples using hierarchical clustering. The analysis package is provided by Spotfire DecisionSite for Functional Genomics 8.1 (Spotfire, Inc., Somerville, MA, USA). Similarity measure was the Euclidean distance, the clustering method was Unweighted Pair Group Method with Arithmetic Mean, and input rank was the ordering function.

Forty-two of the most highly expressed up- or downregulated genes in patients with JRA were used in pathway modeling using PathwayAssist Software (Ariadne Genomics Inc., Rockville, MD, USA). Relationships of protein nodes with H_2_O_2 _and calcium were preserved intentionally to reveal the overall networking of calcium influx and peroxide metabolism, which are highly specific to the function of neutrophils.

Hypervariable (HV) genes are a group of genes whose expressions exhibit higher variation than biological fluctuation baseline, as we have described previously [14]. After the HV genes were selected, they were clustered using an F-means clustering method to determine each gene's cluster association and its connectivity with other genes. Genes were sorted based on their cluster association and connectivity in the control group, with the gene of the highest connectivity of the first cluster ranked on the top. To reveal the intrinsic dynamic relationship between each gene in a sample group, a matrix of correlation coefficiency was displayed in a colour mosaic.

### Polymerase chain reaction validation of array data

Six down randomly selected genes in the patients with polyarticular JRA and controls were selected for reverse transcription-polymerase chain reaction (PCR) confirmation.

#### Reverse transcription

Three controls and three patients were used for PCR validation. First-strand cDNA was generated from 1.2 μg of total RNA per sample with 0.1 ng of the exogenous control *Arabidopsis *RUBISCO mRNA (RCA) spiked in (Stratagene, La Jolla, CA, USA) according to the OmniScript Reverse Transcriptase manual, except for the use of 500 ng anchored oligo dT primer (dT_20_VN). cDNA was purified with the Montage PCR Cleanup kit (Millipore Corporation) according to manufacturer's instructions. cDNA was diluted 1:20 in water and stored at -20°C.

#### Quantitative PCR

Gene-specific primers for the human genes *CD74*, *V-FOS*, *NFKBIA*, *PTGS2*, *SCYA3L1*, *SCYA4*, and the *Arabidopsis *gene RCA were designed with a 60°C melting temperature and a length of 19 to 25 bp for PCR products with a length of 90 to 130 bp, using ABI Primer Express 1.5 software (Applied Biosystems, Foster City, CA, USA). PCR was run with 2 μl cDNA template in 15 μl reactions in triplicate on an ABI SDS 7700 using the ABI SYBR Green I Master Mix and gene-specific primers at a concentration of 1 μM each. The temperature profile consisted of an initial 95°C step for 10 minutes (for Taq activation), followed by 40 cycles of 95°C for 15 seconds, 60°C for 1 minute, and then a final melting curve analysis with a ramp from 60°C to 95°C for 20 minutes. Gene-specific amplification was confirmed by a single peak in the ABI Dissociation Curve software. No template controls were run for each primer pair and no RT controls were run for each sample to detect nonspecific amplification or primer dimers. Average threshold cycle (Ct) values for RCA (run in parallel reactions to the gene of interest) were used to normalise average Ct values of the gene of interest. These values were used to calculate the average group (normal versus patient), and the relative ΔCt was used to calculate fold change between the two groups.

### ELISA for S100 A8/A9

Costar High Binding 96-well plates (Corning Life Sciences, Acton, MA, USA) were coated with 100 μl/well of S100A8/A9-specific monoclonal antibody 5.5 (kindly provided by Dr. Nancy Hogg, Cancer Research UK, London, UK) diluted to a concentration of 1 μg/ml in 0.1 M carbonate buffer (pH 9.6) and left overnight at 4°C. After incubation, the plates were washed with phosphate-buffered saline (PBS)/0.1% Tween-20 and blocked with PBS/0.1% Tween-20/2% bovine serum albumin (BSA) (100 μl/well) for 30 minutes at room temperature. The samples (plasma from children with polyarticular JRA and healthy controls) and standards (100 μl) were added and incubated for 40 minutes at room temperature. After three washes with PBS/0.1% Tween-20, the plates were incubated with 100 μl/well of S100A9 polyclonal antibodydiluted 1:10,000 in PBS/0.1% Tween-20/2% BSA for 40 minutes at room temperature. After incubation, the plates were washed three times and incubated with 100 μl/well of peroxidase-conjugated donkey anti-rabbit immunoglobulin G (IgG) at a dilution of 1:7,500 in PBS/0.1% Tween-20/2% BSA for 40 minutes at room temperature. After three washes, the presence of IgG was detected with 100 μl of a peroxidase substrate solution (3,3',5,5'-tetramethylbenzidine; RDI Division of Fitzgerald Industries Intl, Concord, MA, USA, formerly Research Diagnostics Inc.) according to the manufacturer's instructions; the reaction was stopped by adding 100 μl of 0.36 mM H_2_SO_4_, and the optical density was read at 500 nm. Results from patient samples were compared against standards of known S100A8/A9 concentration. The detection limit for this assay is 1 ng/ml A8/A9 dimer. The antibodies used in this assay have been tested against murine S100A8 and S100A9, bovine S100A and S100B, and human S100A12 and found to be specific.

Results were tabulated in a commercially available statistics and graphics software program (GraphPad Prism; GraphPad Software, Inc., San Diego, CA, USA), and comparisons of children with active and inactive polyarticular JRA and controls were accomplished using a two-tailed independent t test. Results ≤ 0.05 were considered statistically significant.

### Immunofluorescence staining

Neutrophils were placed on glass coverslips, incubated with 1 μg fluorescein isothiocyanate (FITC)-conjugated anti-myeloperoxidase (MPO) at 4°C for 30 minutes, and then washed again with Hanks' balanced salt solution at room temperature. Cells were observed using an axiovert fluorescence microscope (Carl Zeiss, Inc., Thornwood, NY, USA) with mercury illumination interfaced to a computer using Scion image processing software (Scion Corporation, Frederick, MD, USA). A narrow band-pass discriminating filter set (Omega Optical, Inc., Brattleboro, VT, USA) was used with excitation at 485/22 nm and emission at 530/30 nm for FITC. A long-pass dichroic mirror of 510 nm was used. The fluorescence images were collected with an intensified charge-coupled device camera (Princeton Instruments Inc., Trenton, NJ, USA).

### Detection of NAD(P)H oscillations

NAD(P)H autofluorescence oscillations were detected as described [15,16]. An iris diaphragm was adjusted to exclude light from neighboring cells. A cooled photomultiplier tube held in a model D104 detection system (Photon Technology International, Inc., Birmingham, NJ, USA) attached to a microscope (Carl Zeiss, Inc.) was used.

## Results

### Microarray analysis of peripheral blood JRA neutrophils

**Figure 1:**
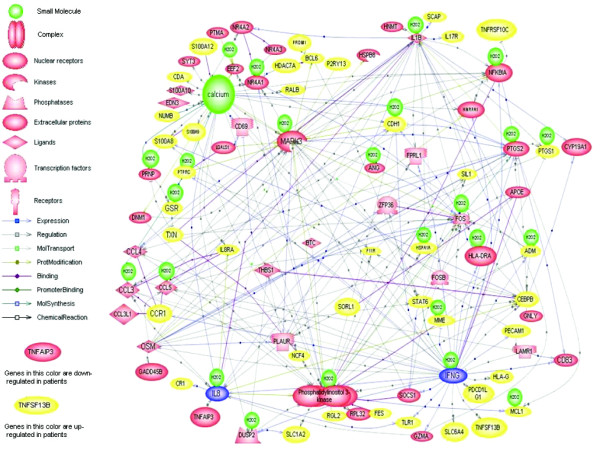
Computer model of differentially expressed genes in juvenile rheumatoid arthritis and control neutrophils developed from PathwayAssist software as described in Materials and methods. Note upregulation of S100 proteins in patients (top left). Also note clusters of genes independently or interdependently regulated by interleukin-8 or interferon-γ (blue circles, bottom left and right). Finally, computer modeling showed significant associations between differentially expressed genes and the regulation of fundamental metabolic processes such as H_2_O_2 _production (multiple green circles) and calcium influx (top left).

A total of 712 genes were shown to have differential levels of expression between the patients with polyarticular JRA and the control subjects. For simplicity, the 84 genes showing the highest levels of differential expression expression are shown in Table 1 (genes over-expressed in polyarticular JRA neutrophils) and Table 2 (genes under-expressed in polyarticular JRA neutrophils). The full data sets are available online [17]. Genes over-expressed in patients with polyarticular JRA included principally mediators and regulators of oxidative response, neutrophil activation, and inflammation control (Table 1) (Figure 1), suggesting that peripheral neutrophils are active in patients with polyarticular JRA and contribute to the systemic inflammatory nature of this disorder. These results provide a catalog of neutrophil-mediated aspects of disease pathology, with both well-characterised and putative pathogenic pathways identified, suggesting that inhibition of neutrophil activity may provide a useful means of limiting key aspects of the pathology of polyarticular JRA. Genes downregulated in JRA neutrophils relative to healthy controls (Table 2) included the immune and inflammatory mediators *CCL3*, *CCL4*, *CCL5*, *IL-1B*, *COX-2*, *MHC-II DR-α*, *granzyme A*, *galectin 1*, *V-Fos*, and inhibitor of nuclear factor-κB-α.

**Figure 2:**
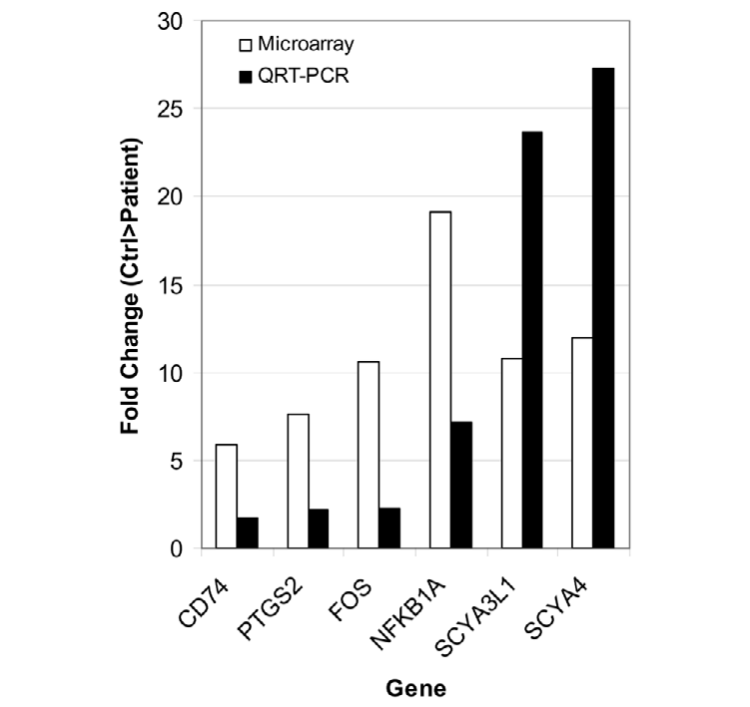
Validation of microarray data with quantitative real-time polymerase chain reaction (QRT-PCR) showing a representative experiment (repeated one additional time). Three controls and three patients were selected for QRT-PCR to validate microarray results. QRT-PCR was carried out for individual samples, and then the average threshold cycle (Ct) of the patients and the average Ct of the healthy controls were used to calculate relative expression, expressed as fold change. The fold changes of both microarray (open bars) and QRT-PCR (solid bars) are shown. For all six genes selected, relative expression was higher in healthy controls relative to patients as shown by microarrays and QRT-PCR, thus confirming the microarray results.

Validity of the array data was then tested using quantitative real-time PCR on the six randomly selected genes (Figure 2). In each case, real-time PCR data corroborated the array findings, as shown in Table 3.

To determine the functional relationship among these genes, computer modeling based on the differentially expressed genes was used. These studies indicated links to both innate and adaptive immunity (Figure 1), with clusters of both interleukin (IL)-8- and interferon-γ (IFN-γ)-regulated genes differentially expressed in children with polyarticular JRA and control subjects. Furthermore, multiple genes in the computer model were linked to both calcium influx (Figure 1, top left) and superoxide ion production (green circles, 'H_2_O_2_'). These findings were of considerable interest given that IL-8 and IFN-γ independently regulate oscillations of key metabolites in neutrophils, which in turn regulate both calcium ion influx and superoxide ion release [18]. This model was tested directly using single-cell autofluorescence, as described below.

### Genomic evidence for persistence of disease activity in JRA neutrophils

Hierarchical clustering of genes that were differentially expressed in patients with polyarticular JRA was used to group individuals who have similar expression profiles in their peripheral blood neutrophils. Patients with polyarticular JRA and controls formed distinct clusters, confirming the validity of the differential gene expression analysis on a global scale.

**Figure 3:**
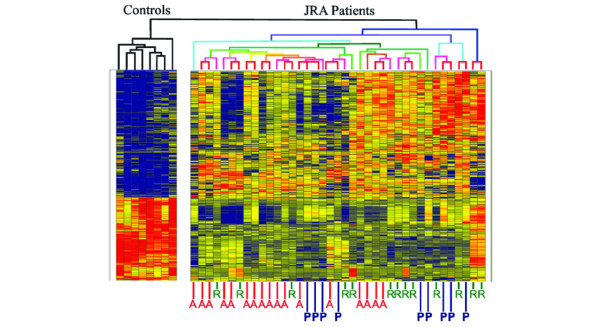
Hierarchical cluster analysis of microarray data in juvenile rheumatoid arthritis (JRA) neutrophils. Data show clustering of control subjects to the left of the grid based on patterns of gene expression. Data of children with JRA are scattered on the right side of the grid regardless of disease status. That is, data of children with active disease (A) cluster together with those of children with partially responsive disease (P) and inactive disease (fully responsive disease) (R).

**Figure 4:**
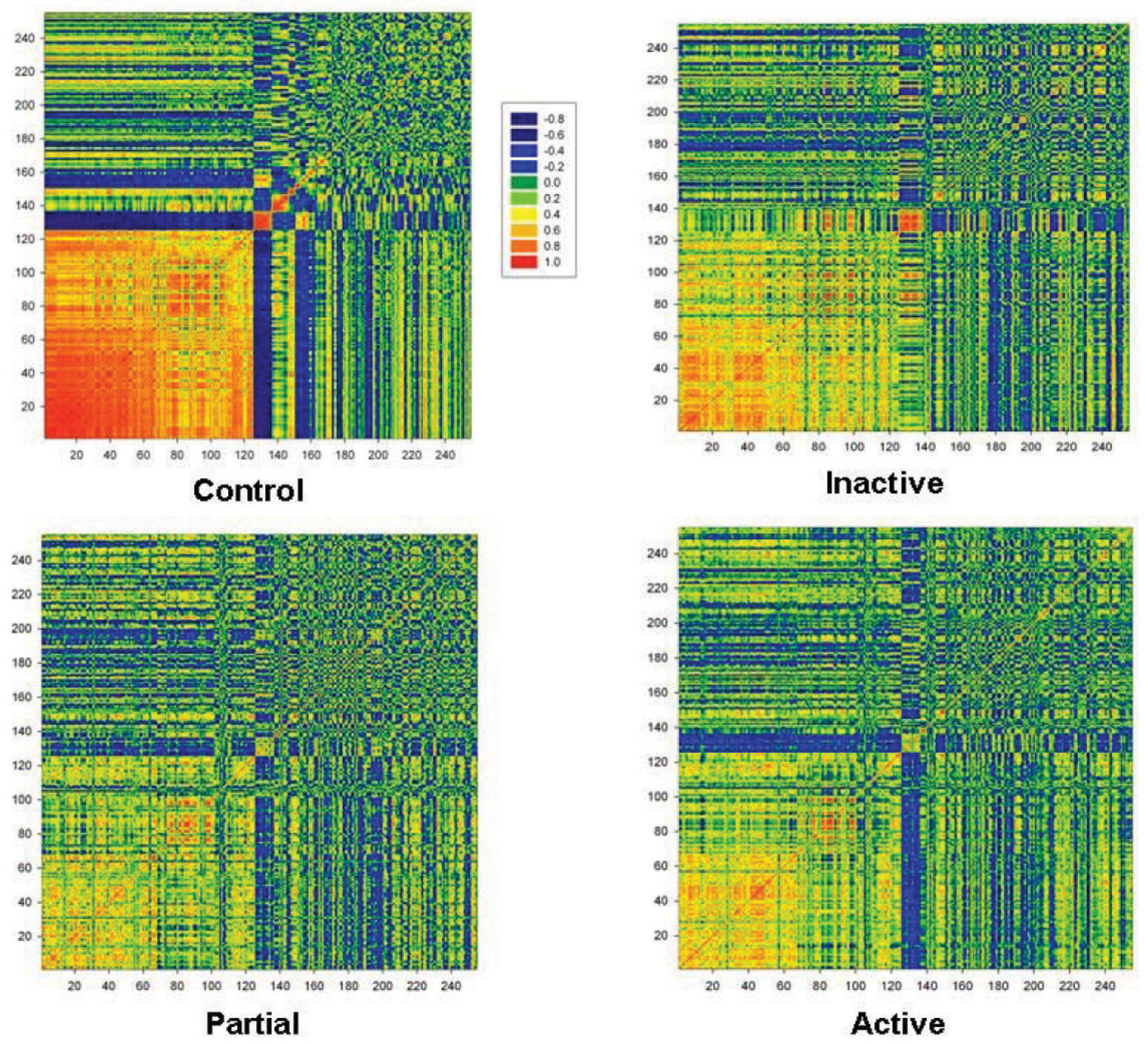
Contingency analyses of neutrophils from children with juvenile rheumatoid arthritis (JRA) and controls. Control samples are represented on the top left panel. Families of genes whose expression levels correlated positively (red) or negatively (green) with one another are displayed on the grid. These same relationships are distorted in neutrophils of children with active polyarticular JRA (bottom right panel) and are only partially restored after full response to therapy (top right panel).

Figure 3 shows a hierarchal cluster analysis of neutrophil mRNA expression in children with polyarticular JRA and a panel of eight healthy control subjects. Children were grouped according to disease activity as described in Materials and methods. Of note is that healthy control subjects cluster together at the left side of the graph. Children with polyarticular JRA, however, scatter across the graph regardless of disease activity. That is, children with polyarticular JRA showed persistent abnormalities in neutrophil gene expression when their disease was well controlled. This finding was similarly demonstrated using the connectivity analysis procedure (Figure 4) described in Materials and methods and in our previously published work [13,14]. The contingency analysis for these selected genes demonstrated disruption of normal gene relationships in neutrophils of children with polyarticular JRA when those relationships were compared with healthy controls. These findings strongly suggest that neutrophils are chronically dysregulated in polyarticular JRA and that therapy only minimally ameliorates the disordered pattern.

**Figure 5:**
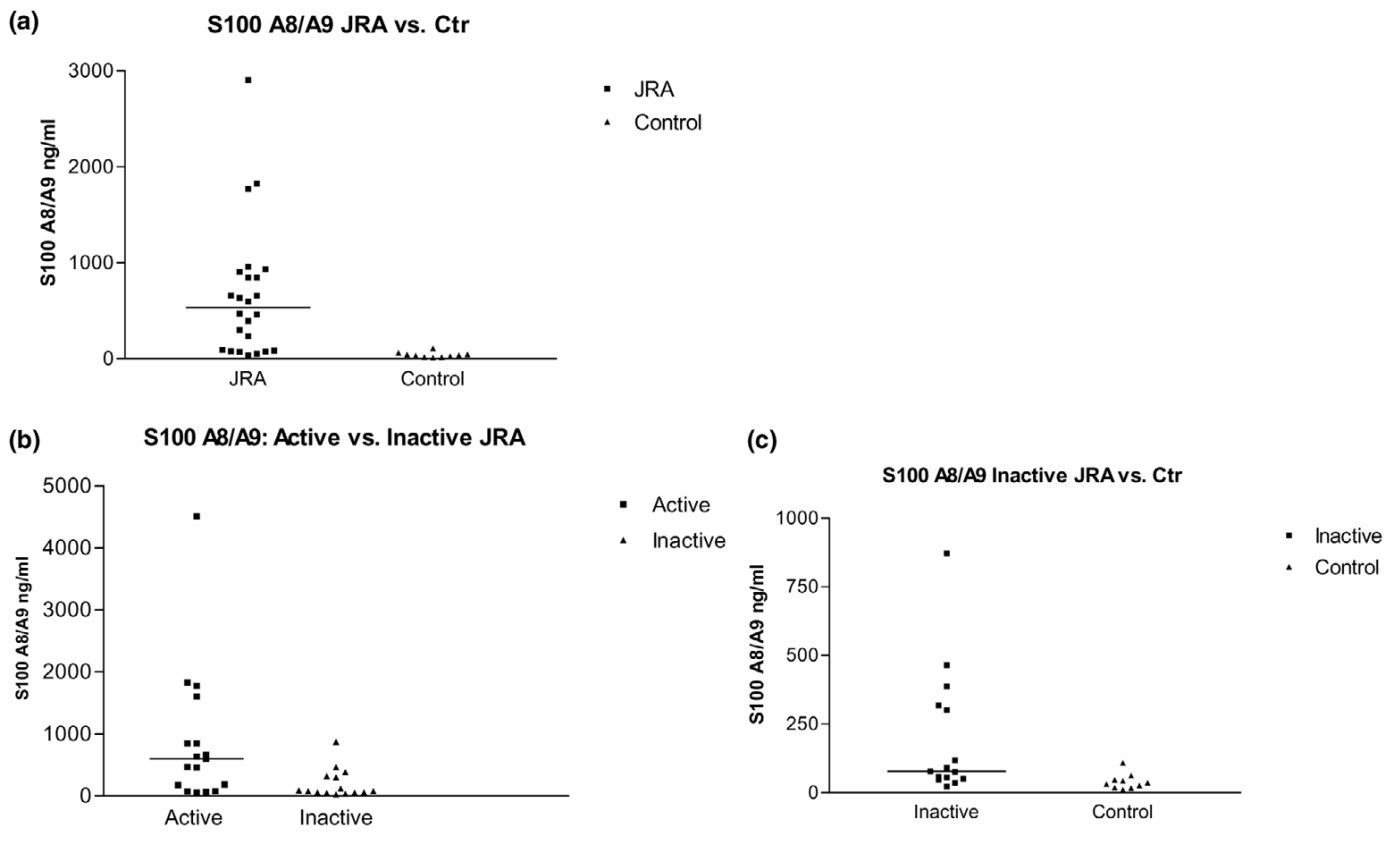
Scatterplots showing plasma levels of S100A8/A9 complexes in children with juvenile rheumatoid arthritis (JRA) and healthy controls (*n *= 10). **(a) **Comparison of all children with JRA (*n *= 24) at the time the initial sample was obtained for analysis. S100 proteins were markedly elevated in children with JRA (662 ± 40 ng/ml) compared with controls (40 ± 9 ng/ml; *p *> 0.001). Although S100A8/A9 levels were higher in children with active disease than with inactive disease (198 ± 60 ng/ml; *p *= 0.007) **(b)**, S100 protein levels were significantly higher (*p *= 0.047) in children with inactive JRA compared with controls **(c)**. Ctr, control.

To further support a role for chronic neutrophil activation in polyarticular JRA, we examined S100A8/A9 and S100A12 plasma levels. Both S100A8/A9 and S100A12 (data not shown) were identified as over-expressed in patients with polyarticular JRA (relative to controls; Figure 1) in array experiments and confirmed on real-time PCR analysis. These proteins are highly expressed in neutrophils and monocytes (up to 40% of cytosolic proteins), are released upon cell activation, and contribute to the migration of neutrophils to inflammatory sites [19,20]. As predicted from the array data, S100 proteins were markedly elevated in children with polyarticular JRA (662 ± 40 ng/ml) compared with controls (40 ± 9 ng/ml; *p *> 0.001; Figure 5a). Children with inactive disease (198 ± 60 ng/ml) had lower levels of S100 proteins compared with children with active disease (*p *= 0.007; Figure 5b), but levels were still significantly higher (*p *= 0.047) than those seen in healthy controls (Figure 5c). These findings suggest that neutrophils in children with polyarticular JRA remain in an activated state during disease quiescence.

**Figure 6:**
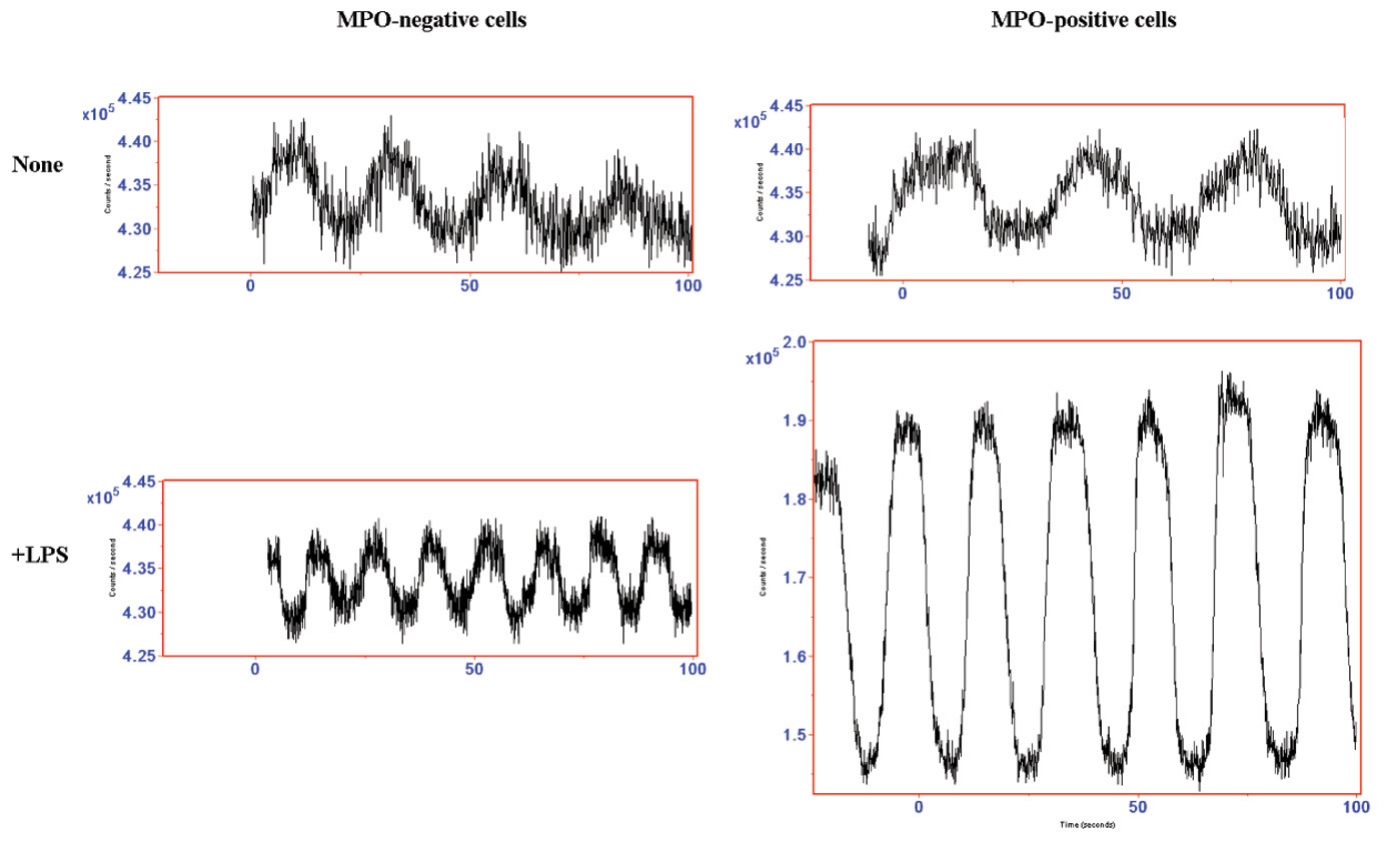
Representative kinetic traces illustrating the JRA-associated abnormality in metabolic oscillations of neutrophils. These traces show NAD(P)H autofluorescence intensity (ordinate) versus time (abscissa); to conserve space, only a few oscillations are shown. Polarised cells were studied on glass slides at 37°C. Using an anti-MPO antibody in immunofluorescence microscopy, JRA neutrophils can be classified as MPO-negative (left) and MPO-positive (right). Untreated MPO-negative cells demonstrated NAD(P)H oscillations with a period of approximately 20 seconds (trace a). The NAD(P)H oscillatory period of these cells decreased to 10 seconds in the presence of the activator LPS. In patients with JRA, a subpopulation of neutrophils are MPO-positive. In the absence of cell stimulation, MPO-positive cells cannot be distinguished from MPO-negative cells. However, in contrast to MPO-negative cells, MPO-positive cells undergo both a decrease in period to 10 seconds and a dramatic increase in the oscillatory amplitude. JDA, juvenile rheumatoid arthritis; LPS, lipopolysaccharide; MPO, myeloperoxidase; NAD(P)H, nicotinamide adenine dinucleotide (phosphate).

The computer model generated through analysis of differentially expressed genes (Figure 1) suggested pathologically relevant links between IL-8- and IFN-γ-regulated genes in polyarticular JRA neutrophils and that gene expression was functionally linked to calcium influx and superoxide ion production. IL-8 and IFN-γ independently regulate oscillatory phenomena in neutrophils, with IFN-γ regulating amplitude and IL-8 oscillatory frequency. We proceeded to test that model by monitoring the autofluoresence of NAD(P)H in living neutrophils, which reflects various stages and mechanisms of neutrophil activation [21]. Metabolic oscillations of neutrophils from six children with polyarticular JRA and five healthy control subjects were monitored. Figure 6 provides representative tracings of NAD(P)H oscillations in resting and stimulated cells from patients. Because metabolic frequencies and amplitudes have been linked with the hexose monophosphate shunt activity and the peroxidase cycle, respectively, we assessed MPO surface expression on living neutrophils. In contrast to controls that show no MPO surface expression, all patients with polyarticular JRA demonstrated a subpopulation of neutrophils (10% to 23% of the cells) that expressed surface-associated myeloperoxidase. Neutrophils staining MPO-negative from patients responded to lipopolysaccharide (LPS) stimulation by increasing the frequency of NAD(P)H oscillations, reducing the period from 20 to 10 seconds, as previously described in activated neutrophils [18]. This behaviour is identical to that observed for control neutrophils. However, MPO-positive neutrophils from patients with polyarticular JRA (including two with inactive disease) demonstrated increases in both frequency and amplitude in NAD(P)H oscillation after LPS stimulation. In contrast, activated control cells show no changes in metabolic amplitude. This novel finding suggests a fundamental breakdown in the regulation of neutrophil metabolism, as will be discussed below. We are now preparing to determine whether the number of aberrantly functioning, MPO-positive cells changes with disease severity or during the course of therapy.

## Discussion

Polyarticular and pauciarticular JRA have long been assumed to be T cell-driven autoimmune diseases [22]. However, involvement of the innate immune system, at least in the polyarticular form of JRA, has long been recognised and is demonstrated by abundant experimental evidence [2-5]. Furthermore, the most successful new therapies for the treatment of polyarticular JRA have been those directed at cytokines released during the innate immune response (that is, TNF-α and IL-1) [23]. Despite this tantalising evidence that innate immunity plays a critical role in the pathogenesis of polyarticular JRA, this aspect of the immune response has been largely overlooked in investigations into basic disease mechanisms.

We demonstrate that neutrophils from children with polyarticular JRA show persistent abnormalities even after the disease has responded to therapy. Furthermore, this observation is supported using multiple measures of neutrophil structure and function. Gene microarrays, plasma S100 protein levels, and single-cell auto fluorescence support the hypothesis that there is a fundamental activation abnormality in neutrophils of children with polyarticular JRA. These studies also demonstrate that multiple methods of analysis applied to gene expression studies can uncover important clues into disease pathogenesis.

We used computer modeling to attempt to unravel the pathogenic clues behind our array data, as we did in a smaller study [13]. Three interesting patterns emerged from that analysis (Figure 1): (a) high levels of mRNA for proteins that regulate neutrophil-endothelial cell interactions (that is, S100A8/A9 and S100A12), (b) large numbers of genes controlling or controlled by superoxide ion production, and (c) genes independently and interdependently regulated by IFN-γ and IL-8. The significance of these findings will be discussed in the following paragraphs.

S100 proteins (also known as calgranulins or myeloid-related proteins) are released from neutrophils during interactions with activated endothelium [24]. Other authors have previously demonstrated that these proteins are elevated in children with both poly- and pauciarticular JRA and have suggested that S100 protein levels may be useful biomarkers, as their levels remain elevated even after other markers of disease activity (for example, erythrocyte sedimentation rate or plasma C-reactive protein) return to normal [25]. Although the clinical utility of measuring S100 protein levels has yet to be demonstrated, we believe that they provide important insights into JRA disease pathogenesis. We have previously proposed that the endothelium represents a critical, and under-investigated, factor in JRA pathogenesis [6]. *In vitro *models, furthermore, support the notion that there are likely to be complex interactions among circulating immune aggregates, leukocytes, and endothelium in polyarticular JRA [26,27], interactions which (in and of themselves) may lead to low-level T-cell activation without the addition of TCR-CD3-transduced signaling [28]. The presence of elevated levels of S100 proteins in polyarticular JRA suggests dysregulation of neutrophil-endothelial cell interactions, but whether the primary abnormality lies in the neutrophils or endothelium cannot be deduced by examining S100 protein levels alone. It is also important to note that S100A8/A9 activates T lymphocytes [29] and could therefore participate in T-cell activation commonly thought to be involved in JRA pathogenesis.

The finding of clusters of IFN-γ- and IL-8-regulated genes differentially expressed in polyarticular JRA neutrophils was of considerable interest, as IFN-γ and IL-8 independently regulate neutrophil oscillatory activities. Oscillatory phenomena are seen on both a macroscopic and microscopic level in biological systems. On the macroscopic level, the most obvious examples would be heartbeat and respiration. However, levels of key metabolites, including superoxide ion and NAD(P)H, also have been shown to oscillate in neutrophils, and these oscillations are causally linked to downstream neutrophil effector functions [30]. Known inflammatory mediators, including TNF-α, IFN-γ, IL-2, and IL-8, regulate these oscillatory phenomena. However, amplitude enhancement and frequency enhancement are controlled by separate, independent, and well-insulated metabolic pathways. IL-8 regulates changes in oscillation frequency, and IFN-γ regulates changes in oscillation amplitude [31]. Thus, the finding that a subpopulation of polyarticular JRA neutrophils exhibit loss of insulation separating the mechanisms that normally regulate amplitude and frequency enhancement is both novel and intriguing. It is important to point out that the defect in metabolic dynamics is contingent upon activation of the hexose monophosphate shunt pathway. That is, there is no defect in JRA until the shunt is activated by LPS or fMLP (*N*-formyl-L-methionyl-L-leucyl-L-phenylalanine) (data not shown). However, when the shunt is activated in polyarticular JRA neutrophils, both metabolic pathways are triggered, which leads to an exaggerated cell response. This process, like S100 protein levels, is likely tied to enhanced secretory activity, in that myeloperoxidase, like S100 proteins, is normally stored in intracellular granules and not released in control cells, although surface expression is seen for some polyarticular JRA neturophils. Precisely how this occurs and how the defect relates (or is related) to the altered expression of IL-8- and IFN-γ-regulated genes are now the subject of investigation in our laboratories.

There are obviously some unanswered questions that emerge from this study. The first is whether the neutrophil defect is primary or secondary and how it relates (if at all) to adaptive immune processes believed to be operative in polyarticular JRA. Previous studies reported from our group [13] support the hypothesis that the defect may be primary. In earlier studies of JRA using whole blood buffy coats, we demonstrated both by discriminant function analysis and connectivity analyses identical to those shown in Figure 4 that JRA gene expression patterns return to normal after response to pharmacologic therapy, regardless of the therapy used. In this larger study using a more robust gene array, we demonstrate that abnormal patterns of neutrophil gene expression persist even when the disease is inactive.

Another question that arises is that of specificity. It is reasonable to ask, for example, whether the same or similar abnormalities of gene expression and neutrophil activation may not be part of other JRA subtypes or any other chronic inflammatory state. The elevation of S100A8/A9 complexes in the serum are clearly not specific to polyarticular JRA, as such elevations have been described in other JRA subtypes [32,33] and other chronic inflammatory diseases [34,35]. However, even if these findings are not specific for polyarticular JRA, they point to important, previously unrecognised contributions of neutrophils in JRA pathogenesis and the need to examine in much more detail than has been previously the case the role of innate immunity in JRA pathogenesis. Despite their limits, our data suggest that there may be pathogenic similarities between polyarticular JRA and chronic autoinflammatory states such as NOMID (neonatal onset multisystem inflammatory disease) [36] and related disorders of neutrophil activation [37].

We have demonstrated through multiple lines of evidence that polyarticular JRA is associated with chronic, dysregulated neutrophil activation. Further investigations into the role of neutrophils in the JRA subset are likely to yield novel and unexpected insights into disease pathogenesis.

## Conclusion

We provide evidence that the neutrophils in polyarticular JRA are in a chronic, activated state. These findings suggest that neutrophils play a critical role in disease pathogenesis.

## Abbreviations

BSA = bovine serum albumin; ELISA = enzyme-linked immunosorbent assay; FITC = fluorescein isothiocyanate; HV = hypervariable; IFN-γ = interferon-γ; IgG = immunoglobulin G; IL = interleukin; JRA = juvenile rheumatoid arthritis; LPS = lipopolysaccharide; MPO = myeloperoxidase; NAD(P)H = nicotinamide adenine dinucleotide (phosphate); OUHSC = Oklahoma University Health Sciences Center; PBS = phosphate-buffered saline; TNF-α = tumour necrosis factor-α.

## Competing interests

The authors declare that they have no competing interests.

## Authors' contributions

JNJ designed the study, enrolled patients, and assisted with data analysis and interpretation. HRP designed and directed the metabolic oscillation studies and assisted in their interpretation. YT performed data analysis and interpretation of the microarray studies. MBF assisted in the development of the gene array used here, directed the labeling and scanning procedure, and assisted in data analysis and interpretation. PAT directed the performance and interpretation of S100 protein ELISAs. ID assisted YT in data analysis and interpretation. KJ directed the cell separation and RNA purification procedures. AK performed the metabolic oscillation studies. YC assisted with sample preparation and RNA purification. CC assisted with primer design and real-time PCR assays. MT assisted with primer design and real-time PCR assays. PS assisted in labeling and scanning of microarrays as well as data analysis and interpretation. JLM assisted with patient recruitment and assignment of disease activity status and developed the database used to record disease activity status and clinical parameters. MC directed hybridisation, scanning, and data analysis activities. All authors read and approved the final manuscript.

## Figures and Tables

**Table 1:** Genes over-expressed in JRA neutrophils

GenBank accession no.	Symbol	Description	Avg. control	Avg. patients	Ratio P/C
NM_001124	ADM	Adrenomedullin	0.3	3.2	10.3
NM_001706	BCL6	B-cell CLL/lymphoma 6 (zinc finger protein 51)	54.0	179.9	3.3
NM_001729	BTC	Betacellulin	0.2	2.8	12.1
NM_001295	CCR1	Chemokine (C-C motif) receptor 1	1.5	5.6	3.7
NM_001785	CDA	Cytidine deaminase	10.9	30.6	2.8
NM_004360	CDH1	Cadherin 1, type 1, E-cadherin (epithelial)	0.3	2.2	6.4
NM_005194	CEBPB	CCAAT/enhancer binding protein (C/EBP), beta	1.1	3.7	3.2
NM_000651	CR1	Complement component (3b/4b) receptor 1, including Knops blood group system	6.6	21.3	3.2
AF172398	F11R	F11 receptor, JAM1	0.5	3.5	7.2
NM_002005	FES	Feline sarcoma oncogene	35.6	108.3	3.0
NM_001462	FPRL1	Formyl peptide receptor-like 1	21.0	72.0	3.4
NM_000637	GSR	Glutathione reductase	3.7	18.5	5.0
NM_015401	HDAC7A	Histone deacetylase 7A	3.3	9.7	2.9
NM_002127	HLA-G	HLA-G histocompatibility antigen, class I, G	336.2	956.2	2.8
NM_005345	HSPA1A	Heat shock 70-kDa protein 1A	20.6	53.7	2.6
NM_014339	IL17R	Interleukin 17 receptor	10.2	28.5	2.8
NM_000634	IL8RA	Interleukin 8 receptor, alpha	13.6	53.9	4.0
BC017197	MCL1	Myeloid cell leukemia sequence 1 (BCL2-related)	5.3	21.5	4.1
NM_007289	MME	Membrane metallo-endopeptidase (neutral endopeptidase, enkephalinase, CALLA, CD10)	9.5	29.5	3.1
NM_013416	NCF4	Neutrophil cytosolic factor 4 (40 kDa)	20.0	58.6	2.9
AF171938	NUMB	Numb homolog (Drosophila)	3.2	11.6	3.7
NM_023914	P2RY13	purinergic receptor P2Y, G-protein coupled, 13, GPR86	8.7	32.0	3.7
NM_014143	PDCD1LG1	programmed cell death 1 ligand, B7-H1	0.3	5.1	15.3
NM_000442	PECAM1	Platelet/endothelial cell adhesion molecule (CD31 antigen)	6.2	12.5	2.0
NM_001198	PRDM1	PR domain containing 1, with ZNF domain	0.8	6.8	8.9
NM_000962	PTGS1	Prostaglandin-endoperoxide synthase 1 (prostaglandin G/H synthase and cyclooxygenase)	0.3	8.6	29.5
NM_002838	PTPRC	Protein tyrosine phosphatase, receptor type, C	55.9	159.7	2.9
NM_002881	RALB	V-ral simian leukemia viral oncogene homolog B (ras related-GTP binding protein)	5.2	15.8	3.0
NM_004761	RGL2	ral guanine nucleotide dissociation stimulator-like 2, RAB2	3.0	8.9	3.0
NM_005621	S100A12	S100 calcium binding protein A12 (calgranulin C)	62.9	164.0	2.6
NM_002964	S100A8	S100 calcium binding protein A8 (calgranulin A)	791.4	2,017.6	2.5
NM_002965	S100A9	S100 calcium binding protein A9 (calgranulin B)	1,152.7	2,697.1	2.3
D83782	SCAP	SREBP CLEAVAGE-ACTIVATING PROTEIN	0.3	2.6	7.5
NM_022464	SIL1	Endoplasmic reticulum chaperone SIL1, homolog of yeast	0.3	5.0	14.9
NM_004171	SLC1A2	Solute carrier family 1 (glial high affinity glutamate transporter), member 2	7.5	32.9	4.4
NM_001045	SLC6A4	Solute carrier family 6 (neurotransmitter transporter, serotonin), member 4	22.8	44.5	2.0
NM_003105	SORL1	Sortilin-related receptor, L(DLR class) A repeats-containing	31.9	89.7	2.8
NM_003153	STAT6	Signal transducer and activator of transcription 6, interleukin-4 induced	1.6	7.9	5.0
NM_003263	TLR1	Toll-like receptor 1	10.4	28.6	2.8
NM_003841	TNFRSF10C	Tumour necrosis factor receptor superfamily, member 10c, decoy without an intracellular domain	8.9	39.6	4.5
NM_006573	TNFSF13B	Tumour necrosis factor (ligand) superfamily, member 13b	9.2	22.4	2.4
NM_003329	TXN	Thioredoxin	9.0	24.8	2.8

Avg. control, average (normalised) intensity in controls; Avg. patients = average (normalised) intensity in patients; Ratio P/C, fold difference between patients and controls.

**Table 2:** Genes under-expressed in patients with JRA

GenBank accession no.	Symbol	Description	Avg. control	Avg. patients	Ratio C/P
NM_001145	ANG	Angiogenin, ribonuclease, RNase A family, 5	18.8	6.1	3.1
NM_000041	APOE	Apolipoprotein E	16.9	7.2	2.3
NM_002983	CCL3	chemokine (C-C motif) ligand 3	166.7	7.4	22.4
D90145	CCL3L1	chemokine (C-C motif) ligand 3-like 1	197.7	21.4	9.2
NM_002984	CCL4	chemokine (C-C motif) ligand 4	221.7	22.2	10.0
NM_002985	CCL5	chemokine (C-C motif) ligand 5	38.5	8.3	4.7
NM_001781	CD69	CD69 antigen (p60, early T-cell activation antigen)	23.5	4.5	5.2
NM_004233	CD83	CD83 antigen (activated B lymphocytes, immunoglobulin superfamily)	24.3	0.1	365.9
NM_031226	CYP19A1	cytochrome P450, family 19, subfamily A, polypeptide 1	25.8	9.2	2.8
NM_004408	DNM1	Dynamin 1	13.6	4.1	3.3
NM_004418	DUSP2	Dual specificity phosphatase 2	54.1	7.8	6.9
NM_000114	EDN3	Endothelin 3	36.7	11.8	3.1
NM_001961	EEF2	Eukaryotic translation elongation factor 2	114.1	29.1	3.9
NM_005252	FOS	V-fos FBJ murine osteosarcoma viral oncogene homolog	483.5	100.4	4.8
NM_006732	FOSB	FBJ murine osteosarcoma viral oncogene homolog B	89.6	11.7	7.7
NM_015675	GADD45B	Growth arrest and DNA-damage-inducible, beta	87.5	19.3	4.5
NM_012483	GNLY	Granulysin	47.5	2.5	19.2
NM_006144	GZMA	Granzyme A (granzyme 1, cytotoxic T-lymphocyte-associated serine esterase 3)	15.3	4.5	3.4
NM_019111	HLA-DRA	Major histocompatibility complex, class II, DR alpha	226.5	33.9	6.7
NM_006895	HNMT	Histamine N-methyltransferase	13.4	4.4	3.1
NM_031157	HNRPA1	Heterogeneous nuclear ribonucleoprotein A1	31.8	8.8	3.6
NM_014365	HSPB8	heat shock 22-kDa protein 8	23.8	7.0	3.4
NM_000576	IL1B	Interleukin 1, beta	169.0	22.4	7.5
AK055991	LAMR1	Laminin receptor 1 (67 kDa, ribosomal protein SA)	35.3	14.2	2.5
NM_002305	LGALS1	Lectin, galactoside-binding, soluble, 1 (galectin 1)	15.5	4.3	3.6
X60188	MAPK3	Mitogen-activated protein kinase 3	228.5	81.3	2.8
NM_020529	NFKBIA	Nuclear factor of kappa light polypeptide gene enhancer in B-cells inhibitor, alpha	705.4	56.0	12.6
NM_002135	NR4A1	Nuclear receptor subfamily 4, group A, member 1	46.0	15.4	3.0
NM_006186	NR4A2	Nuclear receptor subfamily 4, group A, member 2	35.7	9.5	3.8
U12767	NR4A3	Nuclear receptor subfamily 4, group A, member 3	18.3	4.4	4.2
BC011589	OSM	Oncostatin M	19.8	5.3	3.8
NM_002659	PLAUR	Plasminogen activator, urokinase receptor	23.8	4.8	4.9
NM_000311	PRNP	Prion protein (p27-30)	15.0	5.1	3.0
NM_000963	PTGS2	Prostaglandin-endoperoxide synthase 2 (prostaglandin G/H synthase and cyclooxygenase)	174.9	30.4	5.8
NM_002823	PTMA	Prothymosin, alpha (gene sequence 28)	169.0	56.0	3.0
NM_000994	RPL32	Ribosomal protein L32	174.4	58.6	3.0
NM_002966	S100A10	S100 calcium binding protein A10 (annexin II ligand, calpactin I, light polypeptide [p11])	33.9	9.2	3.7
NM_003745	SOCS1	suppressor of cytokine signaling 1, SSI-1	23.1	8.2	2.8
NM_032298	SYT3	synaptotagmin III, DKFZp761O132	30.2	11.4	2.7
NM_003246	THBS1	Thrombospondin 1	33.7	11.1	3.0
NM_006290	TNFAIP3	Tumour necrosis factor, alpha-induced protein 3	112.1	24.9	4.5
NM_003407	ZFP36	Zinc finger protein 36, C3H type, homolog (mouse)	177.8	51.5	3.5

Avg. control, average (normalised) intensity in controls; Avg. patients, average (normalised) intensity in patients; Ratio C/P, fold difference between controls and patients.

**Table 3:** Summary of real-time polymerase chain reaction data

	Fold change (control > patient)		
Gene	Microarray	Polymerase chain reaction	Directional match
*CD74*	5.9	1.7	Yes
*PTGS2*	7.6	2.2	Yes
*V-FOS*	10.6	2.3	Yes
*NFKB1A*	19.1	7.2	Yes
*SCYA3L1*	10.8	23.7	Yes
*SCYA4*	12	27.3	Yes
